# Elevated IgG Antibody to Aluminum Bound to Human Serum Albumin in Patients with Crohn’s, Celiac and Alzheimer’s Disease

**DOI:** 10.3390/toxics9090212

**Published:** 2021-09-04

**Authors:** Aristo Vojdani

**Affiliations:** 1 Immunosciences Lab, Inc., 822 S. Robertson Blvd, Ste. 312, Los Angeles, CA 90035, USA; immunsci@gmail.com or drari@msn.com; Tel.: +1-310-657-1077; 2 Cyrex Laboratories, LLC. 2602 South 24th St., Phoenix, AZ 85034, USA

**Keywords:** autoimmunity, aluminum, adjuvant, neoantigen, Alzheimer’s, Crohn’s, celiac

## Abstract

Aluminum is in our water and food, and is used as an adjuvant in vaccines. About 40% of the ingested dose accumulates within the intestinal mucosa, making the gut the main target of inflammation and autoimmunity; about 1% accumulates in the skeletal system and brain, inducing the cross-linking of amyloid-β-42 peptide and the formation of amyloid aggregates associated with Alzheimer’s disease. To examine whether the accumulation of aluminum in the gut and brain tissues results in neoantigen formation, we bound aluminum compounds to human serum albumin. We used ELISA to measure IgG antibody in 94 different sera from healthy controls and 47 sera from each group of patients: anti-*Saccharomyces cerevisiae* antibody-positive (Crohn’s), and positive for deamidated α-gliadin and transglutaminase-2 IgA antibodies (celiac disease), autoimmune disorders associated with intestinal tissue antigens. Because earlier studies have shown that aluminum exposure is linked to Alzheimer’s disease etiology, and high aluminum content is detected in Alzheimer’s patients’ brain tissue, we also measured aluminum antibody in the blood of these patients. Additionally, we measured aluminum antibody in the sera of mixed connective tissue disease patients who were positive for antinuclear antibodies, and used them as disease controls. We found significant IgG antibody elevation against all three aluminum compounds in the sera of patients with Crohn’s, celiac and Alzheimer’s disease, but not in patients with mixed connective tissue disease. We concluded that aluminum ingestion and absorption from the GI tract and brain may contribute to Crohn’s, celiac and Alzheimer’s disease, but not to mixed connective tissue disease.

## 1. Introduction

The industrialization of the world during the past century has led to the accumulation of aluminum and other heavy metals, not only in our surrounding ecosystems, but in our bodies as well. Due to the demand in developed countries in the past 60 years, the production of aluminum and its use has increased by more than 35% [1,2]. For the general population, the main route of exposure to aluminum is through water, after it has gone through a filtration system, and through processed food, for which aluminum is used for preservation purposes. Aluminum is also used in pharmacological products such as antiperspirants and anti-acids, through which the metal enters the body [3].

The increased consumption of processed foods such as bread, cakes, pastries, ice cream, candies, cheeses, infant formulas, coffee creamer, chocolate and so on, some with aluminum adjuvants for preservation purposes, some stored in aluminum containers, or even both, are just a few examples of sources of oral intake of aluminum [3,4]. “Food industrialization” has resulted in an increased ingestion of aluminum to a so-called tolerable weekly intake of more than 7 mg/kg in many countries, especially in the North American and European populations [2]. Aluminum salts such as aluminum phosphate, aluminum hydroxide and aluminum potassium sulfate have been used in low concentrations [5,6] in many vaccines such as diphtheria, tetanus, *Haemophylus influenzae*, polio, whooping cough and hepatitis B. This is done to increase the antigenicity of the injected bacterial or viral antigens and enhance the activation of both cellular and humoral immune responses, resulting both in higher titers of antibody production and enhanced T- and B-cell immunological memory to the injected antigen.

This enhanced immune response to the vaccine antigens is due to the electrostatic adsorption of aluminum, which has a positive charge, to the surface proteins, which have negative charges [7,8]. The binding of aluminum to the proteins or antigens results in a strong non-specific immune response against the neoantigen, since aluminum is foreign to our system.

Compared to other metals such as sodium [Na^+^], calcium [Ca^++^], and zinc [Zn^++^], aluminum [Al^3+^] has strong positive charges. It firmly binds to metal-binding amino acids such as arginine, histidine and tyrosine. The binding of Al^3+^ to various proteins causes not only their oligomerization but also induces conformational changes that prevent them from being degraded by proteases. Furthermore, Al^3+^ also binds to phosphorylated amino acids, which promotes self-aggregation and accumulation of neurofilament proteins, microtubule-associated proteins and other phosphorylated cytoskeletal proteins [7]. This oligomerization and induction of conformational changes of proteins by Al^3+^ makes this complex of aluminum and proteins [neoantigens] the target of both cellular and humoral attacks, leading to the induction of autoimmunities [9].

All these characteristics of Al^3+^ and its combination with bacterial or viral antigens and some vaccine additives, such as casein, increases the retention time of the antigens at the site of injection, and enhances the interaction of neoantigens with newly-recruited antigen-presenting cells, phagocytosis, and the activation of T-helper cells, resulting in enhanced antibody response to vaccine antigens [10].

In fact, the injection of diphtheria toxoid adsorbed with aluminum resulted in a stronger antibody response than the injection of non-adsorbed antigen [11,12]. However, compared to food and water, which are the main routes of entry into the body, the amount of aluminum that is used in vaccines is very small, and a child who consumes baby formula for just two days receives more aluminum from the powdered milk than from a dose of vaccine, which can contain between 85 (HepB) to 156 (DTaP) μg of aluminum [13].

Aluminum is used in different foods as firming agents, pH-adjusting in bakery products, emulsifying agents in processed cheeses, and food coloring in cakes and decorations. For example, the average commercial muffin contains 28 mg of aluminum [14]; a study by Stahl et al. [3] found that cocoa powder had a higher aluminum concentration [165 mg/kg^+^] than almost all the other foods they analyzed. 

Aluminum has been found to adversely affect the gastrointestinal system, the reproductive system, and particularly the nervous system [8,15,16,17,18,19,20,21,22]. However, the impact of aluminum in the gut was not known until the 2013 study by de Chambrun et al. [15]. 

Because aluminum can be found in a soluble form [citrate] and a particular form [phosphate], these investigators [15] evaluated the effects of both soluble and particular forms of aluminum in murine models of colitis. Their results showed that aluminum worsened the intestinal inflammation of chemical-induced colitis. This was supported by their findings that aluminum increased the intensity and duration of macroscopic and histologic inflammation, colonic myeloperoxidase activity, inflammatory cytokine expression, decreased epithelial cell renewal, and impaired intestinal barrier function. Based on these results, it was concluded that aluminum might be an environmental risk factor for inflammatory bowel disease (IBD) [15].

The neurotoxicity of aluminum has been demonstrated in tissue culture, animal models, and its link with diseases such as encephalopathy, Parkinson’s disease (PD) and Alzheimer’s disease (AD), and chromic renal failure [15,16,17,18,19,20,21,22,23]. In relation to the neurotoxicity of the metal in AD, mounting evidence has suggested the induction of β-amyloid oligomerization by aluminum action as a cross-linker and the integration of aluminum into the amyloid cascade [8]. This integration of aluminum with various cell proteins and the induction of neuronal cell death was demonstrated by a very high degree of co-localization of aluminum in the brain tissue and in the senile plaques of patients with familial AD using fluorescence microscopy and complementary imaging for amyloid-β [18].

Since aluminum acts as a cross-linker to a variety of proteins and forms neoantigens, our aim in this study was to examine immune response to aluminum hydroxide, aluminum citrate and aluminum potassium sulfate bound to human serum albumin (HSA) in sera obtained from blood donors (controls), patients with autoimmune diseases, and patients with a disorder unrelated to aluminum for contrast and control. We chose patients with Crohn’s disease who are positive for anti-*Saccharomyces cerevisiae* antibodies (ASCA) and patients with celiac disease who are positive for α-gliadin and transglutaminase (tTG) antibodies, because these two autoimmune disorders are associated with intestinal tissue antigens, and we wish to study the association of aluminum accumulation in the gut with autoimmune diseases. Because earlier studies have shown that aluminum exposure is linked to Alzheimer’s disease etiology, and high aluminum content is detected in Alzheimer’s patients’ brain tissue, we also measured aluminum antibody in the blood of patients with Alzheimer’s disease who are positive for amyloid-β-peptide (Aβ-peptide) and phosphorylated tau antibodies and had reversed ratio between amyloid-β-40 versus amyloid-β-42 peptides. Finally, we measured aluminum antibody in the sera of patients with mixed connective tissue disease (MCTD) who are positive for antinuclear antibody (ANA), to use as disease controls (see Table 1).

## 2. Materials and Methods

Commercially available sera from 94 nominally healthy blood donors, and 47 sera each from different groups of patients with ASCA positivity/Crohn’s disease, gliadin and tTG positivity/celiac disease, AD, and ANA positivity/MCTD were used for the detection of aluminum antibody.

Commercially available sera of 24 patients with Crohn’s disease and 24 sera from patients with celiac disease were purchased from The Binding Site (San Diego, CA, USA), Inova (San Diego, CA, USA), Trina International (Nanikon, Switzerland), Diamedix (Hialeah, FL, USA), and Innovative Research (Novi, MI, USA). 

We also purchased from Innovative Research additional blood samples obtained from donors who had been screened for anti-*Saccharomyces cerevisiae* antibodies (ASCA), and anti-gliadin and transglutaminase (tTG) IgA for positivity or negativity. We selected 23 samples that had been found positive for ASCA and added them to the Crohn’s patients’ samples for a total of 47. We also selected 23 samples that had been found positive for gliadin and tTG antibodies and added them to the 24 celiac disease patients’ samples for a total of 47.

Sera from 47 Alzheimer’s patients (Caucasian: 37, African-American: 6, Hispanic: 4), 32 males and 15 females, ages ranging from 60 to 82 years, were purchased from Reprocell (Beltsville, MD, USA) and Sanguine BioSciences (Valencia, CA, USA). They were diagnosed according to the National Institute of Neurological and Communicative Disorders and Stroke and the Alzheimer’s Disease and Related Disorders Association (NINCDS-ADRDA) criteria. These sera were used in our earlier study [24].

For antinuclear antibody (ANA) positivity, we screened many samples from blood donors and selected 47 samples with a titer greater than 1:80 for inclusion in this study. 

### 2.1. Preparation of Aluminum-HSA Conjugate

Aluminum hydroxide, aluminum citrate and aluminum potassium sulfate were purchased from Sigma-Aldrich^®^ (St. Louis, MO, USA) One gram of HSA was put in 100 mL of 0.01 M phosphate buffer saline [PBS] at pH 7.4 and was kept on the stirrer for 30 min.

For the binding of aluminum to HSA, 333 mg of each of the three aluminum types was dissolved in 10 mL of 0.01 M phosphate buffer saline [PBS] at pH 7.4. Each aluminum solution was added dropwise to HSA solution over a span of 15 min.

After incubation at room temperature (RT) for 2 h, followed by another 2 h at 37 °C, the mixture was put in a dialysis bag with a cutoff of 6000 against 0.01 M PBS at pH 7.4 for 72 h with a change of buffer every 24 H.

The aluminum-HSA conjugate was centrifuged at 3000× *g* for 15 min, then filtered through a 0.2 micron filter. The binding of the different aluminum types to the HSA was confirmed by gel electrophoresis and shifts in the location of the HSA band. To find the optimal concentration of aluminum-HSA to the ELISA plates, 100 μL of HSA, aluminum-HSA at a concentration of 10 mg/mL, and aluminum salt at 3.3 mg/mL were each diluted 1:100, 1:200, 1:400 and 1:800 in 0.01 M carbonate buffer at pH 9.6 and then added to different duplicate wells of ELISA plates obtained from Corning (Pittston, PA, USA).

Plates were incubated at RT for 6 h, then were put in a refrigerator overnight. The plates were washed 3 times using 0.01 M PBS pH 7.4 containing 0.05% Tween 20, after which 200 μL of 2% bovine serum albumin [BSA] Cohn fraction, which is protease-free, was added to all plate wells. Plates were then kept at RT for 6 h, followed by incubation in the refrigerator for an additional 16 h. The BSA was then removed from the plates, which were washed 3 times using 0.01 M PBS containing 0.05% Tween 20.

### 2.2. Measurement of IgG Antibody to Aluminum-HSA in Human Sera by ELISA

Different sera were added to duplicate wells of each plate coated with aluminum alone, HSA alone and aluminum linked to HSA.

After 60 min incubation at 24 °C, the content was removed, and plates were washed 3 times with 0.01 M PBS pH 7.4 and 100 μL of alkaline phosphatase-labeled anti-human IgG was added to all wells. Following repeated incubation and washing, 100 μL of substrate PNPP at a concentration of 1 mg/mL in substrate buffer was added. 30 min later, the developed color was measured at 405 nm using ELISA reader.

The optical densities (ODs) of each serum sample were recorded after the subtraction of baseline ODs obtained from any reaction of the sera with aluminum or HSA alone.

### 2.3. Specificity of Antigen-Antibody Reaction

#### 2.3.1. Serial Dilution

Four different sera with high levels of IgG anti-aluminum antibody were diluted 1:200, 1:400, 1:800 and 1:1600, and were added to different wells of ELISA plate coated with BSA, or with aluminum hydroxide, aluminum citrate, or aluminum potassium sulfate. After completion of the additional steps for the ELISA assay, the ODs were measured at 405 nm.

#### 2.3.2. Inhibition Assay

Inhibition experiments were performed in liquid phase using the same four serum samples obtained from patients with Crohn’s disease. In different tubes, sera were pre-incubated for 2 h in serum diluent buffer containing 0.1% HSA, (0), 1, 10, 50 and 100 μL/mL of aluminum hydroxide, aluminum citrate, or aluminum potassium sulfate. All tubes were centrifuged at 12,000× *g* for 15 min to remove the antigen-antibody complexes. The supernatants were transferred to aluminum-HSA-coated plates and assessed by ELISA as described above. Inhibition of binding at serum samples preabsorbed by HSA or aluminum was calculated based on the decline in the ODs.

### 2.4. Statistical Analysis

Statistical analysis was performed using GraphPad Prism 6.0 software (GraphPad Software Inc., San Diego, CA, USA). The diagnostic values of the indirect ELISA assays were evaluated by the receiver operating characteristic (ROC) curve. The optimal cutoff values were chosen according to ROC analysis, setting sensitivity respectively for aluminum hydroxide, aluminum citrate, and aluminum potassium sulfate at 0.34, 0.41, and 0.65 for the ASCA+ group, 0.20, 0.56, and 0.81 for the gliadin and tTG IgA+ group, 0.29, 0.53, and 0.63 for the Alzheimer’s group, and 0.16, 0.16 and 0.24 for the ANA+ group. Additional data analysis was performed using STATA 14.2 software (StataCorp LLC, College Station, TX, USA). Logistic regression, t-tests, and Pearson’s correlation coefficients were used to analyze the data. Bonferroni adjustments were performed to avoid a false discovery rate for multiple comparisons.

## 3. Results

### 3.1. Detection of Aluminum Antibody in Blood Donors and in Patients with Different Diseases

We used ELISA plates coated with three different forms of aluminum bound to HSA to measure the presence of aluminum-specific IgG antibody in sera obtained from blood donors and patients who were ASCA-positive, patients who were α-gliadin- and tTG-positive, patients who were ANA-positive, and patients who were positive for Alzheimer’s disease. The antibody positivity was confirmed using commercially available kits that were purchased from Inova Diagnostics and Euroimmun.

**Figure 1:**
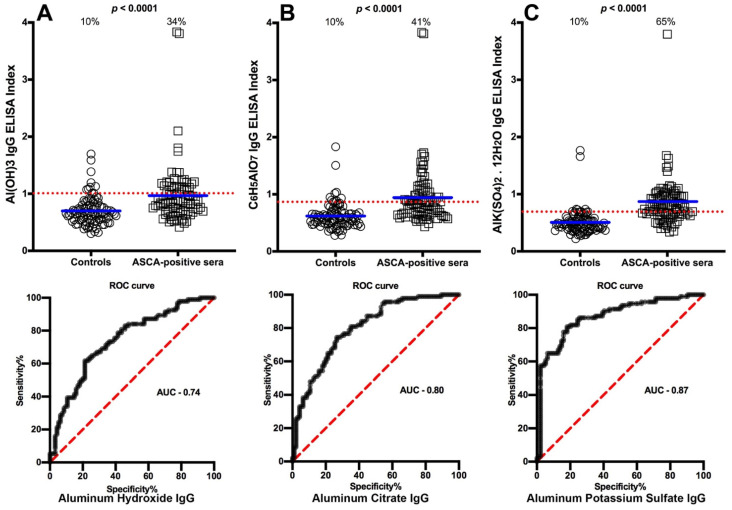
Percentages of samples with elevations in IgG antibodies against three different aluminum compounds in 94 healthy controls and 47 patients positive for ASCA (Crohn’s disease). Dotted horizontal lines indicate the cutoff for positivity used in each assay, as calculated by receiver operating characteristic (ROC) analysis. The percentages of elevation, controls vs. patients, are indicated at the top of each distribution, while the thick short horizontal full bars indicate the respective means (**A**) The percentage of ASCA-positive patients with elevated IgG antibodies against aluminum hydroxide was 34%, with an area under the curve (AUC) of 0.74; (**B**) The percentage of ASCA-positive patients with elevated IgG antibodies against aluminum citrate was 41%, with an AUC of 0.80; (**C**) The percentage of ASCA-positive patients with elevated IgG antibodies against aluminum potassium sulfate was 65%, with an AUC of 0.87.

**Figure 2:**
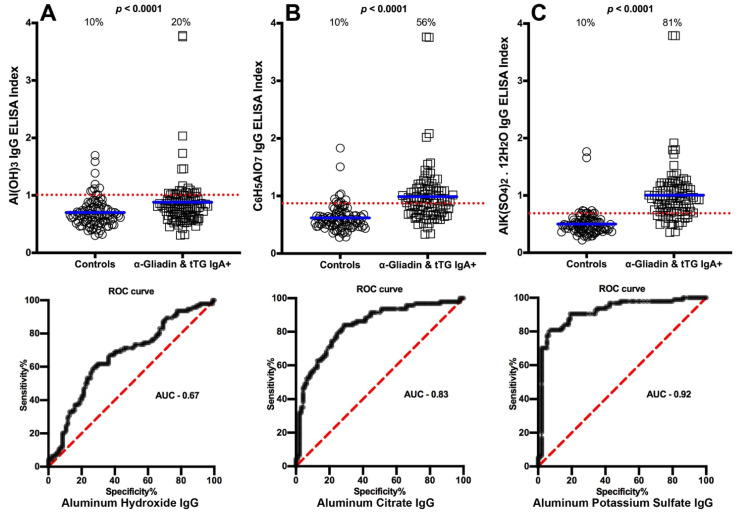
Percentages of samples with elevations in IgG antibodies against three different aluminum compounds in 94 healthy controls and 47 patients positive for gliadin 33-mer peptide and tTG (celiac disease). Dotted horizontal lines indicate the cutoff for positivity used in each assay, as calculated by receiver operating characteristic (ROC) analysis. The percentages of elevation, controls vs. patients, are indicated at the top of each distribution, while the thick short horizontal full bars indicate the respective means (**A**) The percentage of gliadin- and tTG-positive patients with elevated IgG antibodies against aluminum hydroxide was 20%, with an area under the curve (AUC) of 0.67; (**B**) The percentage of gliadin- and tTG-positive patients with elevated IgG antibodies against aluminum citrate was 56%, with an AUC of 0.83; (**C**) The percentage of gliadin- and tTG-positive patients with elevated IgG antibodies against aluminum potassium sulfate was 81%, with an AUC of 0.92.

**Figure 3:**
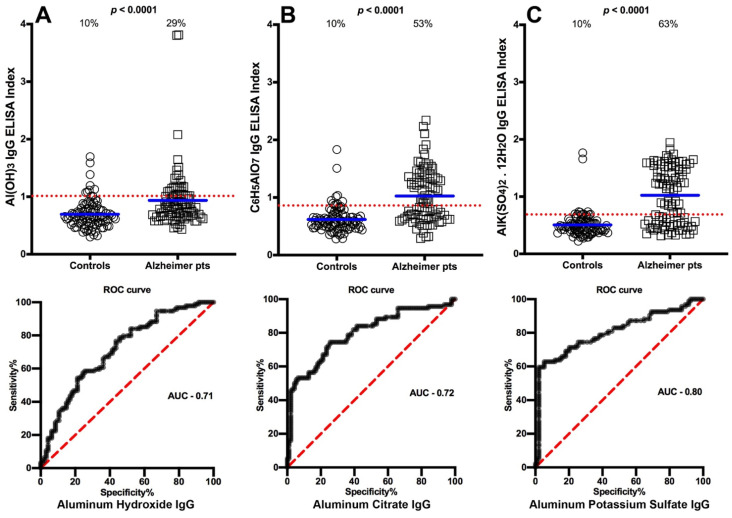
Percentages of samples with elevations in IgG antibodies against three different aluminum compounds in 94 healthy controls and 47 patients positive for Alzheimer’s disease. Dotted horizontal lines indicate the cutoff for positivity used in each assay, as calculated by receiver operating characteristic (ROC) analysis. The percentages of elevation, controls vs. patients, are indicated at the top of each distribution, while the thick short horizontal full bars indicate the respective means (**A**) The percentage of Alzheimer’s disease patients with elevated IgG antibodies against aluminum hydroxide was 29%, with an area under the curve (AUC) of 0.71; (**B**) The percentage of Alzheimer’s disease patients with elevated IgG antibodies against aluminum citrate was 53%, with an AUC of 0.72; (**C**) The percentage of Alzheimer’s disease patients with elevated IgG antibodies against aluminum potassium sulfate was 63%, with an AUC of 0.80.

**Figure 4:**
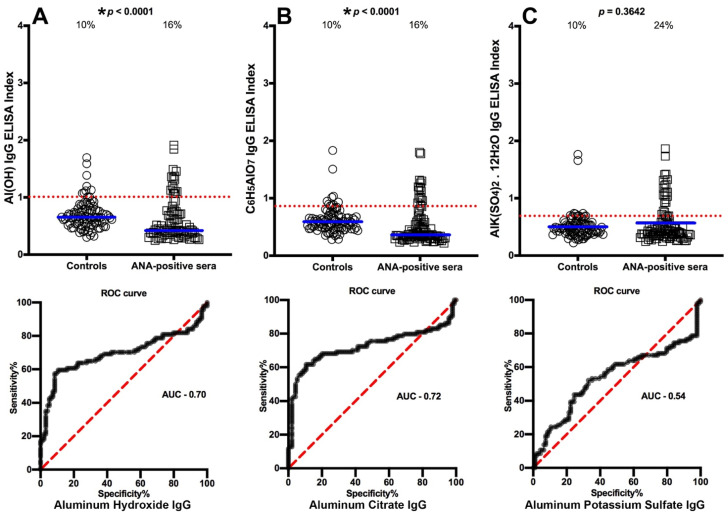
Percentages of samples with elevations in IgG antibodies against three different aluminum compounds in 94 healthy controls and 47 patients positive for antinuclear antibody (ANA) (mixed connective tissue disease [MCTD]). Dotted horizontal lines indicate the cutoff for positivity used in each assay, as calculated by receiver operating characteristic (ROC) analysis. The percentages of elevation, controls vs. patients, are indicated at the top of each distribution, while the thick short horizontal full bars indicate the respective means (**A**) The percentage of ANA-positive patients with elevated IgG antibodies against aluminum hydroxide was 16%, with an area under the curve (AUC) of 0.70; (**B**) The percentage of ANA-positive patients with elevated IgG antibodies against aluminum citrate was 16%, with an AUC of 0.72; (**C**) The percentage of ANA-positive patients with elevated IgG antibodies against aluminum potassium sulfate was 24%, with an AUC of 0.54. * While the *p* values for aluminum hydroxide and aluminum citrate may appear significant (both *p* < 0.0001), this is actually misleading, because the means for these two were actually lower than the means of their controls, which means that the resulting values were actually in reverse or insignificant. The *p* value for aluminum potassium sulfate was clearly not significant (*p* = 0.3642).

We selected OD values that struck a proper working balance between sensitivity and specificity to determine the cutoff points that would differentiate between the levels of antibody made against aluminum in blood donors and in the different patient groups. The percentages of those with elevated aluminum levels and the ROC curve with calculated area under the curve (AUC) in the sera of patients with Crohn’s disease are shown in Figure 1A–C. The data presented in these figures show that while 10% of controls had elevations in IgG antibody to different forms of aluminum, based on cutoff values respectively of 1.010, 0.8655, and 0.6895,the percentage of elevated samples for aluminum hydroxide antibody was 34% (AUC 0.74, *p* < 0.0001), aluminum citrate antibody was 41% (AUC 0.80, *p* < 0.0001), and aluminum potassium sulfate antibody was 65% (AUC 0.87, *p* < 0.0001) as detected in the sera of patients who were ASCA-positive (Crohn’s disease) (Figure 1A–C). In the sera of patients who were gliadin 33-mer peptide- and tTG-positive (celiac disease), based on cutoff values respectively of 1.010, 0.8695, and 0.6895, the percentages were 20% (AUC 0.67, *p* < 0.0001) for aluminum hydroxide, 56% (AUC 0.83, *p* < 0.0001) for aluminum citrate, and 81% (AUC 0.92, *p* < 0.0001) for aluminum potassium sulfate (Figure 2A–C). Based on cutoff values respectively of 1.010, 0.8550, and 0.6895, for the Alzheimer patients the percentages were 29% (AUC 0.71, *p* < 0.0001) for aluminum hydroxide, 53% (AUC 0.72, *p* < 0.0001) for aluminum citrate, and 63% (AUC 0.80, *p* < 0.0001) for aluminum potassium sulfate (Figure 3A–C). In ANA-positive samples (MCTD), the percentages were the lowest at 16% (AUC 0.70, *p* < 0.0001) for aluminum hydroxide, 16% (AUC 0.72, *p* < 0.0001) for aluminum citrate, and 24% (AUC 0.54, *p* = 0.3642) for aluminum potassium sulfate. While the *p* values for aluminum hydroxide and aluminum citrate may appear significant (both *p* < 0.0001), this is actually misleading, because the means for these two were actually lower than the means of their controls, which means that the resulting values were actually in reverse or insignificant. The *p* value for aluminum potassium sulfate was clearly not significant (*p* = 0.3642) (Figure 4A–C).

Our results demonstrated that the percentage of samples with elevated IgG antibodies against aluminum hydroxide bound to HSA was lower than the percentages against aluminum citrate and aluminum potassium sulfate in three of the four patient groups; in the ANA-positive group, the percentage for aluminum hydroxide was equal to aluminum citrate but lower than that for aluminum potassium sulfate.

### 3.2. Specificity of Anti-Aluminum Antibody

The antibody specificity detected in the sera was tested in two separate experiments: serial dilutions and inhibition assays.

Four different sera diluted from 200–1600 did not react with the HSA that was used as a carrier-binding protein. As shown in Table 2, all ODs obtained were very close to the background of the ELISA methodology [0.156–0.231]. However, the same sera reacted with aluminum hydroxide, aluminum citrate, and aluminum potassium sulfate in proportion to their dilutions. For example, the reaction of specific serum with aluminum sulfate at a dilution of 1:200 resulted in an OD of 2.26, and after dilution of 1:1600, the OD was reduced to 0.43 or a reduction of 81% (Table 2). 

In the inhibition study, the addition of different concentrations of aluminum-HSA ranging from 1–100 μg/mL resulted in a dose-dependent inhibition ranging from 2.5–77% for different concentrations of aluminum hydroxide, 3–80% for aluminum citrate, and 13–85% for aluminum potassium sulfate (Table 3). There was no inhibition when HSA alone was added to the liquid phase of the assay. With the other three sera with significant elevations in aluminum antibody, preabsorptions of the sera with aluminum showed inhibition of anti-aluminum antibody binding to aluminum-coated plates from 4–77%. This inhibition in antigen-antibody assay was proportionate to the concentration of aluminum-HSA used as the inhibitor (Table 3).

## 4. Discussion

In this report, we describe the production of antibodies against three different forms of aluminum bound to HSA in the sera of blood donors and patients with ASCA positivity, gliadin and tTG positivity, AD and ANA positivity.

Our results demonstrated that the percentage of samples with elevated IgG antibodies against aluminum hydroxide bound to HSA was lower than the percentages against aluminum citrate and aluminum potassium sulfate in three of the four patient groups; in the ANA-positive group, the percentage for aluminum hydroxide was equal to aluminum citrate but lower than that for aluminum potassoum sulfate.

Compared to blood donors, we detected high levels of IgG antibody against the different aluminum-HSA complexes foremost in samples from patients who were ASCA IgA-positive (an indication of Crohn’s disease), and also in the samples from patients who were positive for α-gliadin-33 mer and tTG IgA (an indication of celiac disease). Although less significant, the percentage of samples with elevated antibodies against HSA-bound aluminum was actually higher in the blood of patients with AD. In comparison, the percentage elevation for patients who were ANA-positive (an indication of MCTD; this was confirmed by 43 of the 47 ANA+ samples testing positive for RNP) was the lowest. For example, while at the specific cutoff point the percentage elevation for IgG antibody against aluminum citrate in controls was 10%, the same percentage for blood samples from patients with ASCA positivity, gliadin and tTG positivity, AD, and ANA positivity was, respectively, 41, 56, 53 and 16 (Figure 1B, Figure 2B, Figure 3B and Figure 4B). These results may indicate that the gastrointestinal tract and the brain are targets for aluminum bound to various proteins containing amino acids, such as arginine, histidine, tyrosine, and phosphorylated AA, which contributes to the formation of neoantigens that may in turn become targets for the immune system and subsequent antibody production [7,8,10,15,18,25].

Although it has been shown that aluminum is an excellent cross-linker of proteins, and that all humans are exposed to it mainly through food and water but also through pharmaceuticals, cosmetics, vaccination, and even systemic allergen immunotherapy [1,2,3,4,23], at the time of this writing our best efforts could find no research article that dealt specifically with the detection of antibody to aluminum after its binding to human tissue proteins, causing conformational changes that result in neoantigen formation [8].

Aluminum can enter the body through different routes, and because of their differences these routes should not be considered automatically equal or proportionate when assessing the immunological impact of the aluminum that passed through them. Inhaling and ingesting aluminum compounds are not the same as injecting them. Vaccines and antigens made for desensitization may contain only micrograms of aluminum, while food, water and medication can deliver milligrams of the metal into the body [25,26]. One might suppose, then, that the micrograms of aluminum conveyed through a vaccine injection would have less of an impact. However, injected aluminum bypasses the protective barriers of the GI tract and mucus membranes, entering directly into the muscle, where it can enter the blood stream and lymph nodes, and from there get distributed to various tissues, including the lungs, spleen, liver, and the brain [8,15,23,24,25,26,27,28,29]. Thus, the administration of micrograms of aluminum into the muscle may have similar toxic effects as ingesting milligrams of the metal [30,31].

In this study we used three different aluminum compounds: aluminum potassium sulfate from food, aluminum citrate from water and aluminum hydroxide, which is used mainly in vaccines and allergens. We bound the aluminum compounds to human serum albumin [HSA] for two reasons: first, chemical haptens only induce immune reactions against a neoantigen after binding to a carrier protein; second, about 34% of transported aluminum manages to bind to albumin [32]. Because the main method of exposure to aluminum for the general population is through food and water, we measured IgG antibody in the blood of patients with two different autoimmune diseases, Crohn’s and celiac disease, since in an animal model of colitis, oral administration of aluminum resulted in deleterious effects on intestinal inflammation and enhanced intestinal permeability [15]. The observed increase in intensity of macroscopic and histologic inflammation, inflammatory cytokine expression, and myeloperoxidase activity may be due to the accumulation of 40% of the ingested dose of aluminum within the intestinal mucosa, with the gut serving as the main storage organ for the metal [19,33].

This 40% buildup of aluminum in the intestinal tissue may be relevant to Crohn’s and celiac disease, since the metal has been identified within macrophages in Peyer’s patches and around the dilated submucosal lymphatics in mesenteric lymph nodes [MLN] [33,34,35,36]. In another study, aluminum was shown to enhance bacterial-induced colitis in mice [37]. Figure 1 and Figure 2 show significant elevation in IgG antibody against aluminum-HSA in sera from both ASCA-positive (Crohn’s) and gliadin- and tTG-positive (celiac disease) patients. This elevation could be related to the accumulation of ingested high doses of the metal in the intestinal mucosa. However, more studies with animal models of Crohn’s and celiac disease are needed to support an association between aluminum accumulation in mucosal tissue and aluminum antibody detection in the blood.

Because earlier studies have shown that aluminum accumulates in the brain [18,38,39], aluminum induced aggregation of bovine brain cytoskeletal proteins in vitro [7], and aluminum has been linked with AD, Parkinson’s disease and autism [8,30,31,32,34,35,36,37,38,39,40,41], we also measured aluminum antibody in the sera of patients with AD. Similar to sera from the ASCA-positive and gliadin- and tTG-positive patients, we found significant elevations in antibodies against all three forms of aluminum used in this study (Figure 3). Finally, we measured aluminum-HSA antibody in sera positive for ANA (MCTD), finding some elevations in the antibodies to these three types of aluminum but in a much lower percentage of the tested sera. In fact, the mean values for aluminum hydroxide and aluminum citrate in the MCTD group were lower than the means for the controls, rendering their mutual *p* value of <0.0001 meaningless, while the *p* value for aluminum potassium in that group was insignificant (Figure 4).

This elevation in IgG antibody to aluminum compounds in patients with Crohn’s, celiac and Alzheimer’s disease, which is an indication of body burden of aluminum, may justify chelation therapy to remove chronic aluminum intoxication [42].

## 5. Conclusions

We have shown that aluminum, after ingestion or injection, can bind to human tissue proteins, resulting in the formation of neoantigens that elicit an antibody immune response.

Our study had the following limitations: we did not measure aluminum levels in the sera collected from the different patient groups; we did not measure aluminum contents in the intestinal and brain tissues of the patients; and we do not know whether the produced antibodies against various aluminum complexes are protecting against or exacerbating the toxicity and harmful effects of aluminum.

We believe our results allow us to make an association between significant elevation in IgG antibody to aluminum-HSA in patients with Crohn’s, celiac and Alzheimer’s disease, and the high degree of aluminum accumulation in the intestinal tissue and brain. More studies are needed to clarify such an association.

## Figures and Tables

**Table 1:** Diseases, corresponding biomarkers, and acronyms.

Diseases	Biomarkers	Acronyms
Crohn’s disease	Anti-*Saccharomyces cerevisiae* antibodies	ASCA
Celiac disease	Transglutaminase	tTG
Alzheimer’s disease	Amyloid-β-peptide	Aβ-peptide
Mixed connective tissue disease	Antinuclear antibody	ANA

**Table 2:** Reaction of serially diluted sera with HSA and aluminum-coated ELISA plates.

Antigens	1:200	1:400	1:800	1:1600
HSA	0.231	0.186	0.178	0.156
Alum hydroxide	1.84	1.19	0.76	0.31
Alum citrate	1.97	1.240	0.86	0.322
Alum sulfate	2.26	1.45	0.92	0.43

The other three sera showed similar declines in antigen-antibody reaction in proportion to dilution.

**Table 3:** Dose-dependent decrease in anti-aluminum binding to aluminum-coated plates.

Antigens	HSA[0] μg/mL	1 μg/mL	10 μg/mL	50 μg/mL	100 μg/mL
Aluminum hydroxide	1.97	1.92	1.37	0.77	0.46
% inhibition	–	2.5	31	61	77
Aluminum citrate	2.17	2.11	1.63	0.94	0.43
% inhibition	–	3	25	57	80
Aluminum sulfate	2.38	2.06	1.45	0.83	0.37
% inhibition	–	13	39	65	85

The other three sera showed similar inhibition of antigen-antibody reaction in pro-portion to the concentrations of aluminum-HSA in the liquid phase of the reaction.

## Data Availability

Additional data is available upon request by contacting the author.
